# Point-of-care SARS-CoV-2 serological assays for enhanced case finding in a UK inpatient population

**DOI:** 10.1038/s41598-021-85247-w

**Published:** 2021-03-12

**Authors:** S. J. C. Pallett, S. J. Denny, A. Patel, E. Charani, N. Mughal, J. Stebbing, G. W. Davies, L. S. P. Moore

**Affiliations:** 1 Centre of Defence Pathology, Royal Centre for Defence Medicine, Queen Elizabeth Hospital Birmingham, Mindelsohn Way, Edgbaston, Birmingham, B15 2WB UK; 2 Clinical Infection Department, Chelsea and Westminster NHS Foundation Trust, 369 Fulham Road, London, SW10 9NH UK; 3 North West London Pathology, Fulham Palace Road, London, W6 8RF UK; 4 NIHR Health Protection Research Unit in Healthcare Associated Infections and Antimicrobial Resistance, Imperial College London, Hammersmith Campus, Du Cane Road, London, W12 0NN UK; 5 Department of Surgery and Cancer, Imperial College London, Hammersmith Campus, Du Cane Road, London, W12 0NN UK

**Keywords:** Clinical microbiology, Infectious-disease diagnostics, Virology

## Abstract

Severe Acute Respiratory Syndrome coronavirus 2 (SARS-CoV-2) has become a global pandemic. Case identification is currently made by real-time polymerase chain reaction (PCR) during the acute phase and largely restricted to healthcare laboratories. Serological assays are emerging but independent validation is urgently required to assess their utility. We evaluated five different point-of-care (POC) SARS-CoV-2 antibody test kits against PCR, finding concordance across the assays (*n* = 15). We subsequently tested 200 patients using the OrientGene COVID-19 IgG/IgM Rapid Test Cassette and find a sensitivity of 74% in the early infection period (day 5–9 post symptom onset), with 100% sensitivity not seen until day 13, demonstrating inferiority to PCR testing in the infectious period. Negative rate was 96%, but in validating the serological tests uncovered potential false-negatives from PCR testing late-presenting cases. A positive predictive value (PPV) of 37% in the general population precludes any use for general screening. Where a case definition is applied however, the PPV is substantially improved (95.4%), supporting use of serology testing in carefully targeted, high-risk populations. Larger studies in specific patient cohorts, including those with mild infection are urgently required to inform on the applicability of POC serological assays to help control the spread of SARS-CoV-2 and improve case finding of patients that may experience late complications.

## Introduction

On 31 December 2019 alarm over a pneumonia-like viral illness caused by an unknown pathogen was first raised to the World Health Organisation. A genome sequence for the presumed causative agent since designated Severe Acute Respiratory Syndrome coronavirus 2 (SARS-CoV-2) was publically released by early January 2020^1^, enabling molecular diagnostics to be rapidly developed. SARS-CoV-2 has now extensively spread and been designated a global pandemic.

Clinical presentation ranges from mild upper respiratory tract infection to severe pneumonia with acute respiratory distress syndrome^2^. The burden on healthcare systems has been devastating, particularly for intensive care services^3^. As of April 2020 there had been over 1 million cases with a case fatality ratio varying from 1 to 6%^4^. The true case fatality ratio is thought to be closer to 1–2% with a considerable number of unrecognised subclinical cases likely to be found in the community. Without the availability of serological testing, this has proven very difficult to accurately assess^2^. While real-time PCR is available during the acute infection it is limited by laboratory and skilled operator requirements^5^, and can only be used early in the symptomatic clinical course. Serologic testing for SARS-CoV-2 offers the potential for enhanced case finding for patients presenting later in the disease course, as well as enabling serosurveillance at a much wider level than molecular diagnostics allow.

Multiple lateral flow serology tests have recently been developed for rapid, point-of-care (POC) use, providing qualitative results within 15 min. Whilst many of these devices have their initial development in China, they require verification in the cohort intended for use before extensive uptake. We take five of these POC kits and evaluate their suitability and potential utility for use in a western European population that had experienced symptoms in line with the Public Health England (PHE) case definition for testing^6^ against the current gold standard of RT-PCR. We go on to take one assay forward for further evaluation (OrientGene COVID-19 IgG/IgM Rapid Test Cassettes), completing 200 assays and commenting on test performance metrics in a UK inpatient population.

## Results

### Validation of serology POC tests

**Figure 1:**
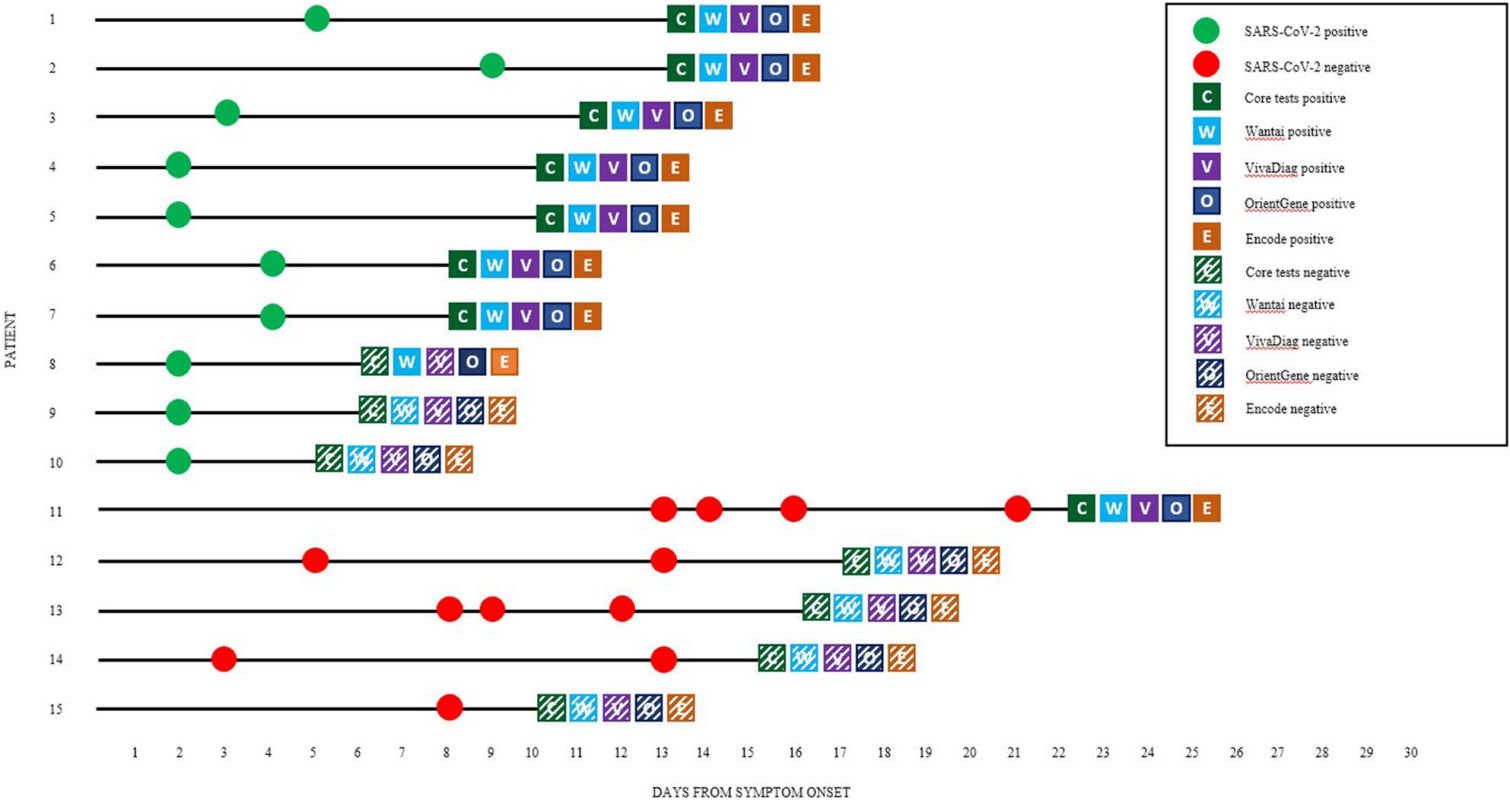
Chronological relationship between onset of symptoms and test results. Results of PCR vs five different point-of-care serological assays among patients meeting the SARS-CoV-2 PHE clinical case definition, March–April 2020, London.

Fifteen patients (mean age 69 years, range 52–85 years, 8/15 female) underwent testing with each of the five assays. All patients had RT-PCR testing conducted between 2 and 10 days from symptom onset, except patient 11 who was first tested at day 13 (Fig. 1; chronological relationship between symptom onset, presentation to secondary care, RT-PCR testing and antibody testing). Of the 75 tests, all were included in the final analysis. No test kit failures were observed. The control line was visible on all 75 test kits run at 15 min post-sampling.

### Validation PCR positive patients

Ten patients with positive RT-PCR SARS-CoV-2 results were tested with each of the five assays (total = 50). The same seven patients were reactive across all five serology tests (35/50; Fig. 1) and were in concordance for reactive tests from day 8 onwards. One further patient was reactive using the SARS-CoV-2 total antibody test (Wantai) (1/50), reactive for IgM only with the COVID-19 split IgG/IgM Rapid Test Cassette (OrientGene) and SARS-CoV-2 split IgM/IgG One Step Rapid Test Device (Encode) at day 6 (2/50) but negative for the other two assays (2/50). The remaining tests were unreactive, all of which were at an earlier stage post symptom onset of day 5 or 6 (10/60).

### Validation PCR negative patients

Five patients with a minimum of one negative RT-PCR result (range 1–4 negative PCRs) were tested with each of the five assays (total = 25). The same four patients were unreactive across all five serology tests (20/25). The fifth patient was reactive using all five assays (5/25).

### OrientGene COVID-19 IgG/IgM rapid test cassette analysis

200 patients (mean age 61 years, range 32–93 years, 43% female) underwent testing. All 200 assays were included in the final analysis. Two test kit failures were observed (failure of solution to ascend chromotography paper), of which both were repeated with subsequent valid reads.

### OrientGene analysis PCR positive patients

**Figure 2:**
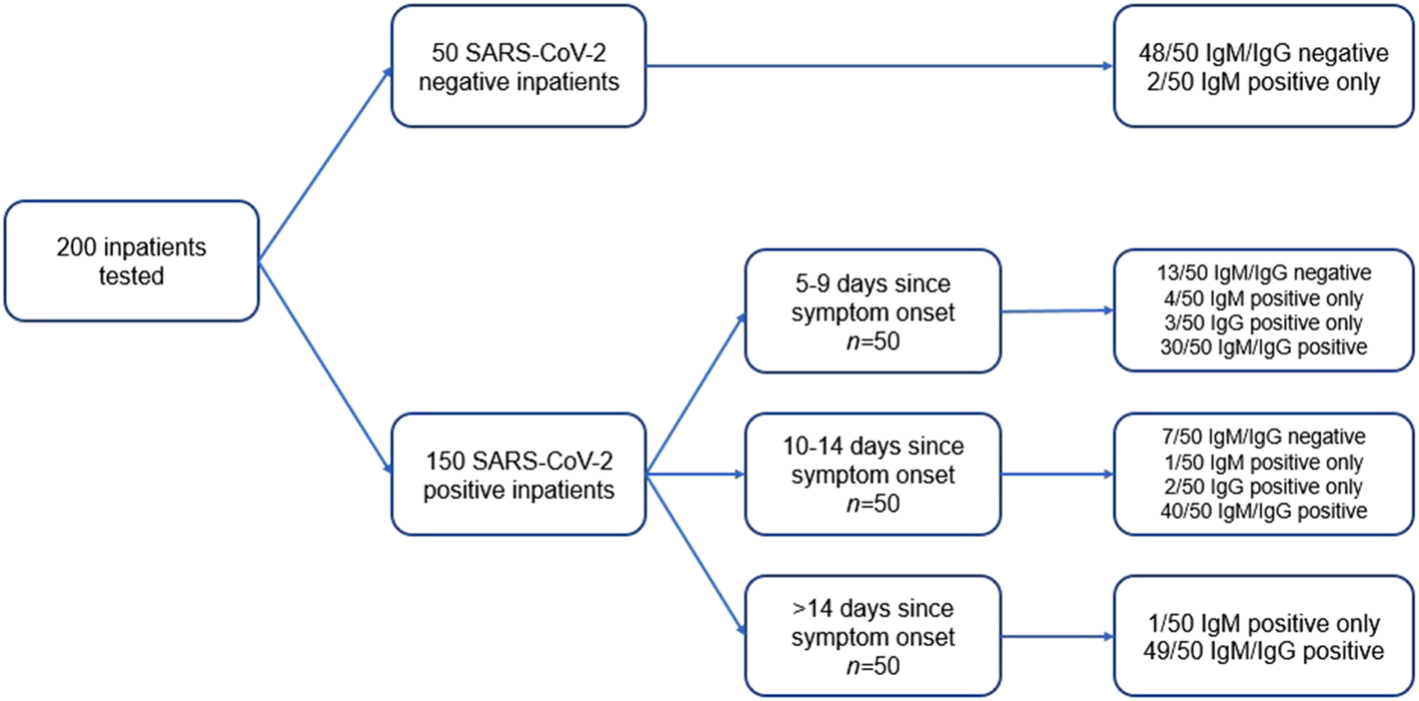
Flowchart demonstrating patient recruitment and subsequent SARS-CoV-2 PCR and serological status. All recruited individuals were inpatients at the time of testing. All patients had a screening chest x-ray as part of the admission protocol and specific comments on findings, or absence of, associated with SARS-CoV-2 infection. None of the 50 negative arm patients had radiological findings consistent with probably SARS-CoV-2 infection. All patients in the positive arm were considered to have moderate to severe SARS-CoV-2 infection, and had radiological findings that were in keeping with probable SARS-CoV-2 infection.

**Figure 3:**
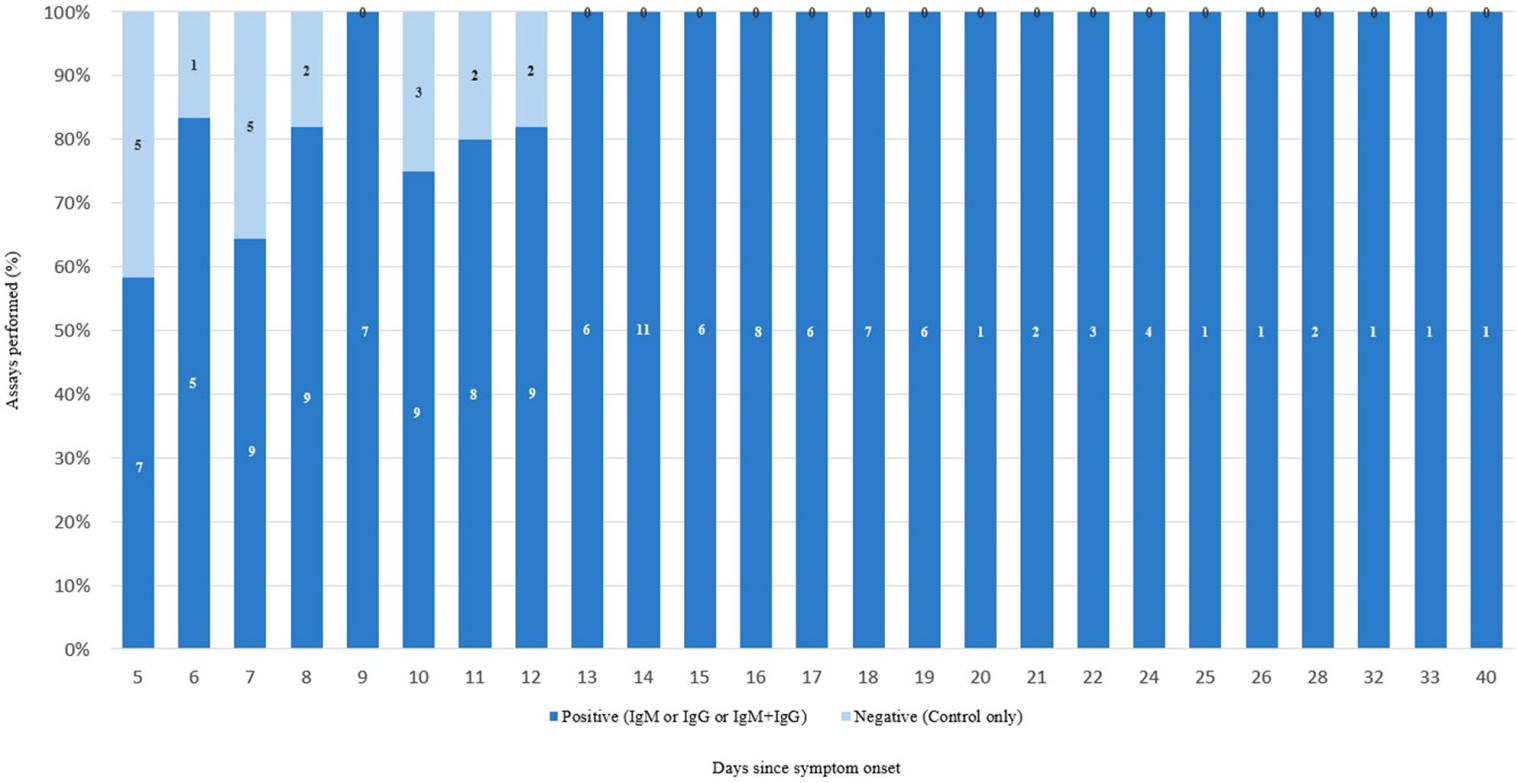
OrientGene point-of-care SARS-CoV-2 serology testing (*n* = 200) among inpatients, March–April 2020, London. Assay results against the number of assays performed on each day post-symptom onset.

150 patients had positive RT-PCR SARS-CoV-2 results (Fig. 2). A total of 130/150 OrientGene COVID-19 split IgG/IgM assays were reactive. Of those tested at 5–9 days post-symptom onset (*n* = 50) 37/50 were reactive (74% sensitivity, 95% confidence interval [CI] 59.7 to 85.4%). At days 10–14 post-symptom onset (*n* = 50), 43/50 were reactive (86% sensitivity, 95% CI 73.3 to 94.2%). At greater than 14 days post-symptom onset (*n* = 50) there were 50/50 reactive assays (100% sensitivity, 95% CI 92.9 to 100%) (Table 1b,c). Tests were uniformly reactive from day 13 onwards (*n* = 67) (Fig. 3). Reactive assays were observed with a single reactive IgM line (6/130), single reactive IgG line (5/130), or both reactive IgM and IgG lines (119/130) (Table 1a). The longest period post-symptom onset tested was at 40 days and demonstrated persistence of both an IgM and IgG reactive line (Fig. 3).

**Table 1:** OrientGene point-of-care SARS-CoV-2 serology testing (*n* = 200) among inpatients, March–April 2020, London.

SARS-CoV-2 PCR	Days post symptom onset	OrientGene serology results			
		CXM	CGX	CGM	CXX
**(a)**					
Positive	5–9	4	3	30	13
	10–14	1	2	40	7
	> 14	1	0	49	0
Negative	N/A	2	0	0	48
		8	5	119	68

	SARS-CoV-2 PCR positive	SARS-CoV-2 PCR negative			
**(b)**					
OrientGene test positive	130	2			
OrientGene test negative	20	48			
Whole cohort sensitivity = 87%; negative rate = 96% (*n* = 200)					

Days post-symptom onset	*n*	Sub-group sensitivity (%)			
**(c)**					
5–9	50	74			
10–14	50	86			
> 14	50	100			

(a) Control, IgM, and IgG line detection across different symptom durations, where *C* control line only, *G* IgM line observed, *M* IgM line observed.

(b) Sensitivity and negative rate across all 200 assays.

(c) Sensitivity and negative rate across different symptom durations.

### OrientGene analysis PCR negative patients

50 patients that were presumed negative (admitted with no symptom or radiological features of SARS-CoV-2 infection and with a negative SARS-CoV-2 PCR test) were included (Fig. 2). Patients were acute admissions under trauma and orthopaedics (12), general surgery (19), burns and plastic surgery (8) and general medicine specialties (11; gout (1), complications of diabetes (2), following a fall (3), palpitations (1), alcohol withdrawal (2), chest pain (1) and gastroenteritis (1)). Assays on 48/50 negative patients were completely unreactive (negative rate 96%, 95% CI 86.3 to 99.5%). Two patients demonstrated a reactive IgM line only.

### POC assay performance characteristics

Re-testing of patients with reactive assays with a second test had demonstrable reproducibility (*n* = 3). The tests with too little blood (*n* = 1), or too much blood (*n* = 1) failed, with no reactivity seen on test lines in the former and no reactivity in test or control lines in the latter.

Where an estimated prevalence of 2.7% in the general population is applied the positive predictive value (PPV) is calculated at 37%^7^, negative predictive value (NPV) 99.6%. When calculated for suspected cases meeting the PHE criteria using a prevalence of 48% (86/177 positive in those tested across the week of 23 March–29 March 2020) in our inpatient population at the same time point there is a PPV of 95.4% and NPV of 88.4%.

## Discussion

Serologic testing for SARS-CoV-2 has the potential to augment control strategies by improving our understanding of the true burden of infection during the current pandemic. While the small number of tests performed (in comparison to the estimated UK prevalence of 2.7% as of 28th March 2020)^6^ during this early validation study makes it difficult to draw high-powered conclusions, the results offer useful insights into the potential utility of POC serological testing at this early stage in the pandemic response. Key concerns exist around the reliability of serology testing and identifying any potential time points for appropriate use.

Our study raises significant concern for the reliance on serology testing in the initial infection phase. Analysis of the larger 200 patient cohort shows that when the initial patient group (days 5–9 post symptom onset) are considered, there is a sensitivity of 74%, with the potential therefore to miss a significant number of positive cases during their most infectious period^7^. Practically, if considering potential use of the assays in the early phase of infection (as suggested by some of the assay manufacturers, and during which period self-isolation is advised by PHE) the sensitivity drops to 66% (*n* = 32). It is not until the end of the second week from symptom onset that the test appears to demonstrate a more practical use. In the greater than 14 day since symptom onset group there was 100% sensitivity. The latest false negative was demonstrated on day 12 with reactive results produced in all tests taken thereafter (*n* = 67) suggesting that there could be real utility in POC serology from 14 days onwards. Where PCR testing is currently limited due to demand this could therefore have a potential role in testing those healthcare workers who have already self-isolated because of possible infection in the last few months. In addition the assay may have a role in community testing where timelines in patients with mild symptoms already self-isolating would be less time critical. A role is not seen however for an impact on public health guidance around self-isolation practices during the infectious period. A PPV of 37% for the general population precludes any consideration of deployment of serology testing for general screening. Where a case definition is applied however, as is required for meeting the PCR testing criteria, the PPV is substantially improved (95.4%) and supports use of serology testing in carefully targeted populations. While limited to detection in targeted populations this would still enable a far better understanding of the infection-fatality ratio then we are currently able to draw from predominantly hospital cases of infection.

At a time when there is considerable pressure on healthcare services it is important to be able to identify SARS-CoV-2 negative patients, streamline pathways and help guide clinical decision making for differential diagnoses. In the larger, second half of the study, serological testing of ‘negative’ patients (n = 50) demonstrated a negative rate of 96% suggesting an additional role in supporting suspected inpatients that return negative PCR results. In particular this could help guide management of patients presenting outside the reliable detection period for PCR swabbing of the upper respiratory tract^7^. If the ‘negative’ patients across both halves of the study (total *n* = 55) are considered*,* three had reactive results, with only one demonstrating a reactive IgG line*.* Given our selection of this ‘negative’ patient from among those who had an a priori clinical suspicion of SARS-CoV-2 infection but who were PCR negative, it is possible or indeed likely that these represented missed cases of SARS-CoV-2. This particular patient presented in extremis with acute respiratory distress-like features (Fig. 1, patient 11). Where PCR is currently the only diagnostic test in use, the ‘missed’ cases tested with PCR raise the possibility of supporting diagnostic capability via serology testing in specific cases. In particular, this may be beneficial in patients presenting during the late phase where PCR is less sensitive or where there is a protracted or unclear history of onset and clinical suspicion is supported by other findings, such as typical radiological changes.

When considering use in outpatient settings, it is vital that a suitable time period is established for a true negative result. Our study suggests that this point is to be found at day 13 post-symptom onset at the earliest, limiting the utility of these assays in enabling return to work or in informing additional self-isolation practices. There is however a real role in helping to identify those healthcare workers and so better understand overall the overall burden of infection in high-risk groups, that have been through a self-isolation period with presumptive infection supported by concomitant reinforcement of advice around the stringent infection, prevention control measures.

If widely employed, demand on suppliers for POC serological assays will be high and interchangeability of kits may therefore be required. The plurality of kits currently available makes it difficult to comment on individual assay performance at this early stage and it is therefore essential to evaluate performance across the range of kits available both against the gold standard (PCR) as well as against each other as soon as possible. Of the five different assays we evaluated, there was direct concordance for detection of SARS-CoV-2 infection across seven of the PCR positive patients (35/38 of reactive results). All five kits failed to detect SARS-CoV-2 infection from two known early positive samples taken at day 5 and day 6 post-symptom onset. Our findings suggested that results consistently appeared reactive from only day 8 post-onset of symptoms onwards across all five kits (extended to day 14 onwards in the larger second half of our evaluation). We cite ongoing concern for their reliability during that early period and the significant impact that missing a positive infection could have on public health control measures.

All POC serology kits are qualitative and while there was general correlation with colour intensity as time from symptom onset increased, variance in intensity of positive results was noted. The test kit instructions comment clearly on expectation for varied colour intensity of positive results and advise that even very weak colour should be considered to be reactive. When considering the practicalities of widespread testing, clear instruction on interpretation will have to feature in standard operating procedures and should allow for comment on interpreting the colour intensity of reactive samples. Antibody levels analysed via validated laboratory-based enzyme-linked immunosorbent assay (ELISA) platforms have suggested higher levels following severe compared to mild infections and cohort studies will now be needed to evaluate the extent of reliability for POC serology testing in mild community infection^8^.

Little in the way of conclusion can be drawn from the five test kits used to explore concerns around end-user difficulties. Re-sampling of reactive patients provided re-producible results and further investigation should be conducted to comment on reproducibility of POC serology kits. Of significant use however is the observation that tests completed against manufacturer protocol by transferring either too little, or too much whole blood into the well resulted in failure. Those tasked with carrying out testing must complete the test as instructed using the marked pipette in order to have reliable results. Failure to register a control line in these cases should avoid the mistake of reporting an inappropriate false result.

When comparing the utility of the SARS-CoV-2 split IgM/IgG tests (Core tests, OrientGene, VivaDiag, Encode) versus the SARS-CoV-2 total antibody test (Wantai), the former have a potential capacity to provide greater information. Of the total reactive tests in the 200 cohort study (*n* = 130), 119 showed both IgM and IgG reactivity. There were 11 reactive samples that differentiated between IgM (6/130) and IgG (5/130) (Table 1a). IgM/IgG reactivity was seen as early as day 5 post-symptom onset and as late as day 40 but there was insignificant data to make conclusions on the IgM/IgG relationship in SARS-CoV-2 infection at this stage. At present we have very little understanding of the immunological response to SARS-CoV-2 and the timeline for which IgM is likely to remain detectable. Of note, subsequent work has demonstrated follow-up testing of IgM only results with laboratory-based anti-nucleocapsid and anti-spike (anti-receptor binding domain) immunoassays shows only limited seroconversion of these cases to IgG and should therefore be interpreted with caution^9^. If SARS-CoV-2 has ongoing transmission among humans, detection of IgM may then have a role in detecting sentinel cases during subsequent periods of infection. Staged evaluation of IgM and IgG relationship in known SARS-CoV-2 positive patients over a prolonged period will be required to inform on the potential additional benefits, if any, of splitting out the IgM and IgG reactivity in POC assays.

Cross-reactivity leading to false positive results is of considerable concern, both with human seasonal coronaviruses and zoonotic beta-coronaviruses, including SARS and MERS. Okba et al*.* used serum samples from 3 SARS-CoV-2 positive patients and found cross-reactivity with the SARS-1 S and S1 proteins, and less so but also with MERS-CoV S protein^8^. The S1 protein appears to be more specific to the SARS coronaviruses, with cross reactivity not seen against an array of PCR positive samples for example seasonal human coronavirus OC43 as well as non-CoV pathogens, including EBV and CMV. Subsequent testing with IgA and IgG specific ELISA showed some cross-reactivity with OC43 PCR positive samples and highlights the importance of assay design, where perhaps multiple protein targets may be required to ensure specificity for SARS-CoV-2^8^. This urgently needs investigation in the western European population before widespread role out of POC SARS-CoV-2 serology. The role of serology as an adjunct to PCR has also recently been reported, with a strong correlation seen between IgM and IgG and the severity of disease^10^. Seroconversion for detection of IgM and IgG was seen at median day 12 and 14 respectively with possibility of detection much earlier in the clinical course and is supportive therefore of our findings of improving sensitivity through the first 14 days of infection^10^.

If shown to be successful in the community setting POC serology testing could have significant implications for tackling the pandemic in resource poor settings which lack the required infrastructure for centralised molecular techniques. The benefits of utilising point-of-care testing to improve the efficiency of case identification and resource allocation in such circumstances has previously been discussed during the Ebola epidemic and could therefore be of considerable use during the SARS-CoV-2 pandemic^11^.

The small number of assays evaluated in this study limits the degree to which conclusions can be drawn. Formal power calculations have not been conducted, but with a sensitivity of 100% at greater than 14 days, accompanied by a margin of error of only 0.04, this exploratory study could now inform larger scale appropriately powered studies to fully characterise the utility of these assays in specific population groups. A greater number of samples are now required in order to comment reliably on sensitivity and specificity and in turn, once the evolving prevalence becomes clearer, the positive and negative predictive value of these tests in various groups. It would also be useful to conduct specificity testing using pre-pandemic serum in order to better assess possible cross-reactivity. The need for rapid initial verification of these assays to augment clinical and public health interventions in this pandemic does not yet enable comment on many aspects of the assays, including the IgM/IgG relationship in the weeks and months post-infection. Awaited longitudinal studies will allow evaluation of assay performance at extended points in the convalescent period and better define an appropriate testing window. Additionally, where results are qualitative and vary in strength of response, it is unclear how test sensitivity would differ when (i) healthcare professionals not used to evaluating lateral flow devices or (ii) the general public read results. Subsequent work has since provided a model for reading SARS-CoV-2 serological assays however which may help with reduce any loss of sensitivity from reading by those less experienced^12^. While our cohort groups include immunocompromised patients, pregnant individuals, and elderly patients, we are unable to sufficiently comment on reliability in these groups. This study is limited to evaluating reactivity in symptomatic adults with moderate-severe disease only and response in children has not been investigated. All positive patients in this study were considered to have moderate-severe infection by nature of the admitting policy of the hospital and evaluation in those with mild disease symptoms is needed to comment further on the population level applicability of these assays.

## Conclusion

SARS-CoV-2 has resulted in a global pandemic and is associated with considerable morbidity and mortality. Serology testing is easy-to-use at the point-of-care and its utility to support, and replace where appropriate, PCR could allow for a significant improvement in the understanding of the true infection burden. This will be vital to the control of further SARS-CoV-2 spread.

Serology testing appears to be unreliable during the early infectious period, however there may be a limited role for use of delayed-case identification in the appropriate context, such as in any patient that is at least 14 days post-symptom onset. Our data would suggest an inferiority to PCR testing in the acute phase of infection, making them inappropriate for use in diagnosis during the infectious period. Further evaluation on a much larger scale is now necessary in order to reliably comment on the earliest stage post symptom onset at which testing can be utilised, as well as on its performance in different population groups. In the meantime, any use of rapid serological test kits must be interpreted with caution, particularly if symptoms are of 14 days or less duration.

## Methods

A prospective single-centre study was completed at a 430-bedded acute London hospital during March 2020. Consecutive inpatients were selected for testing based on real-time PCR detection of SARS-CoV-2 (AusDiagnostics, Australia) and stratified based on duration of symptoms. Patients were included if they were admitted to hospital with symptoms matching the PHE case definition for testing^13^, and had an accurate history recorded of at least 5 days of symptoms.

Exclusion criteria included all patients under 16 years old. Mild infection not requiring admittance to hospital were excluded. Patients that were unable to provide an accurate history or those with vague onset of symptom duration were also excluded in order to accurately report time-lines.

15 SARS-CoV-2 CE marked split IgM/IgG antibody tests (Core tests) were available for paired testing alongside 15 SARS-CoV-2 CE marked total antibody rapid tests (Wantai), 15 COVID-19 CE marked split IgM/IgG Rapid Tests (VivaDiag), 15 COVID-19 CE marked split IgG/IgM Rapid Test Cassettes (OrientGene), and 15 SARS-CoV-2 CE marked split IgM/IgG One Step Rapid Test Devices (Encode). Ten PCR positive and five PCR negative patients were selected and paired serology testing was carried out by the same three doctors (SJCP, AP, SJD) experienced in the use of rapid diagnostic test kits. In order to evaluate reliability of test reactivity in positive patients, five positive patients were evaluated between days 5–9 of symptom duration and five patients between days 10–14. Additionally, five patients thought to be true negative ((i) symptoms meeting the case definition lasting at least 5 days but no more than 10 days at presentation, (ii) at least one negative SARS-CoV-2 PCR result, and (iii) an alternative clinical diagnosis for their symptoms) underwent paired testing in order to assess for false positive reactivity.

Each patient was tested by the same doctors, as per each product protocol. Test kits were then read at the designated time period as per individual protocol and a result recorded for both the appearance of a control line and the presence or absence of antibody test reactivity. All results were verified independently by two of the doctors.

A power-calculation for assessing significance of sensitivity analysis (with an estimate of 90%) was applied in analysis, setting type I error at 0.05 and type II error at 0.20. Assuming assays performed as per the manufacturer information it was calculated that testing 132 PCR positive individuals would be required. Using the same inclusion criteria a further 200 patients were evaluated using the COVID-19 IgG/IgM Rapid Test Cassettes (OrientGene). Patient history from both the Emergency Department assessment and the admission clerking were carefully evaluated and patients excluded if there was doubt over exact symptom onset, including those patients with vague history of onset, presenting with confusion, or following early intubation with an incomplete history. Patients were stratified based on consecutive PCR positive results to one of 3 time interval groups since symptom onset, encompassing 83% of SARS-CoV-2 positive cumulative inpatients at the time of the study period end-point. 50 PCR positive patients were stratified to the 5–9 post-symptom onset group, 50 patients to the 10–14 day post-symptom onset group and 50 patients at greater than 14 days since symptom onset. An additional 50 patients were selected with a presumed negative diagnosis with a negative SARS-CoV-2 PCR result, admitted with an acute surgical complication or had a fever with an alternative, non-respiratory primary diagnosis.

Prevalence estimates at the time of the study, provided by modelling by the MRC Centre of Global Infectious Diseases Analysis report^6^, were used to estimate a PPV if these assays were to be deployed for use in the general population.

Five additional test cassettes were used to evaluate possible anticipated end-user difficulties. Three test cassettes were employed to retest a patient with a positive result to comment on reproducibility. One test cassette was completed with too little blood, failing to fill the pipette to the indicated line and one test cassette was completed with too much blood, by over-filling the well.

### Consent

This study was approved by the Chelsea & Westminster NHS Foundation Trust COVID testing committee. The study was reviewed by the Chelsea & Westminster NHS Foundation Trust Research and Development Office and deemed a verification of a CE marked in vitro diagnostic test, therefore the need individual informed consent was waived. Aggregated data was analysed in accordance to the UK Secretary of State for Health and Social Care general notice that under the Health Service Control of Patient Information Regulations (2002) patient data for a COVID-19 purposes may be used for research. The study was conducted in accordance with relevant guidelines and regulations including the Declaration of Helsinki.

## Data Availability

The data analysed during the current study and further details on the assays are available from the corresponding author (LSPM; l.moore@imperial.ac.uk) on reasonable request, as long as this meets local ethical and research governance criteria.
